# Gigahertz femtosecond laser-by a novel asymmetric one-dimensional photonic crystal saturable absorber device with defect layer

**DOI:** 10.1515/nanoph-2022-0145

**Published:** 2022-05-10

**Authors:** Chun-Yu Song, Hua-Long Chen, Yong-Jie Wang, Liang Jin, Ying-Tian Xu, Lin-Lin Shi, Yong-Gang Zou, Xiao-Hui Ma, Yu-Feng Song, Cong Wang, Ya-Ting Zhang, Ja-Hon Lin, He Zhang, Han Zhang, Jian-Quan Yao

**Affiliations:** State Key Laboratory of High Power Semiconductor Lasers, Changchun University of Science and Technology, Changchun 130022, P. R. China; School of Precision Instruments and Opto-Electronics Engineering, Tianjin University, Tianjin 300072, P. R. China; International Collaborative Laboratory of 2D Materials for Optoelectronics Science and Technology of Ministry of Education, Institute of Microscale Optoelectronics, Shenzhen University, Shenzhen 518060, P. R. China; Institute of Electro-Optical Engineering, National Taipei University of Technology, Taipei 10608, Taiwan

**Keywords:** harmonic, mode locking, photonic crystal

## Abstract

High repetition frequency (HRF) ultrashort pulse fiber laser has been widely used in laser cold processing. The technical solutions such as short cavity length fiber laser have been proposed to achieve HRF ultrashort pulse output recently. However, the application of material-based saturable absorbers in this field has been astricted due to the low modulation depth, low damage resistance threshold, and high saturation fluence. Here, we designed a one-dimensional asymmetric photonic crystal with defect layer (1D-APCDL) as a novel saturable absorber, where the defect layer is Bi_1.6_Sb_0.4_Te_3_ with high modulation depth. The harmonic pulse with 3.82 GHz repetition frequency is achieved at the wavelength of 1562 nm, which is the highest repetition frequency of the topological insulator-based ring fiber laser so far to the best of our knowledge. The research provides a new saturable absorber solution, and provides a new idea for the application of material-based nonlinear optical chip in high-repetition frequency ultrashort pulse fiber lasers.

## 1 Introduction

High repetition frequency femtosecond laser has important applications in optical communication, frequency comb, high-speed optical sampling, and arbitrary waveform generation by virtue of its advantages of low noise and high stability [1–3]. Femtosecond laser also has a pivotal role on account of the demand in fine machining of micro–nano structures, such as optical fiber manufacturing, material etching, and integrated circuit manufacturing, etc. [4–6]. The sample suffers minimal damage due to the short pulse duration. Research suggests that the ablation rate of femtosecond laser machining is 4–6 orders of magnitude higher than the ion beam technique [7]. However, the surface roughness of the sample cannot reach the suitable optical quality at high power, such as the processing of hard materials (mullite ceramics and semiconductors) [8–10]. A strategy to address this issue has been demonstrated that increasing the frequency of the femtosecond laser and reducing the energy of single pulse is effective [11, 12]. Short cavity length mode-locked fiber laser has been widely studied because of their high repetition frequency [13–15]. However, GHz-level high repetition frequency mode-locked fiber lasers require a cavity length of less than 0.2 m, which is obviously difficult at present because of the limitations of the device connection method and the concentration of erbium-doped fibers [16]. Harmonic mode-locking (HML) is one of the solutions to this challenge, which is to achieve multipulse operation by controlling the parameters of the fiber laser cavity [17]. According to the area theorem of optical pulses, the peak power limiting effect leads to the splitting of quantised soliton pulses while the pump power increases significantly, further increasing the repetition frequency of mode-locked pulses [18]. It is worth mentioning that all these operations are carried out without reducing the cavity length. Therefore, obtaining high repetition frequency by harmonic mode-locking is a good choice.

There are two main techniques to exploit HML up to now, i.e., passive mode-locking based on all-fiber structure or real saturable absorber, respectively [19–21]. Among them, the nonlinear polarization evolution (NPE) technique based on the all-fiber structure has been extensively investigated. Xu et al. realized the 22 GHz HML by adjusting dispersion and nonlinearity [22]. However, the NPE is extremely sensitive to external condition. Changes in temperature stress and strain can affect the nonlinear effects of nonlinear polarization evolution, limiting its application in complex environments [23]. Although the gain depletion and recovery (GDR) mechanism has been proposed in recent years, which can theoretically suppress the jitter of the harmonic pulse in the all-fiber structure. However, most of the related work is based on numerical simulation and only a few related experiments have been reported [24]. Material-based mode-locked fiber laser has attracted enormous attention by virtue of its high stability. It is considered to be a means of implementing HML with greater resistance to interference than NPE [25]. Grudinin et al. combine the two mechanisms. Multi-quantum-well (MQW) semiconductor structure saturable absorber and nonlinear amplifying loop mirror (NALM) were designed in the ring cavity to achieve harmonic pulses exceeding 2 GHz [26]. However, MQW structures usually need to be fabricated by means of epitaxial growth equipment, which significantly increases the cost of the device.

The past decade has seen a growing trend towards to explore nano materials, which have been demonstrated to achieve mode-locking as saturable absorbers [27–30]. Extensive research has shown that real saturable absorbers (SAs) with high nonlinearity and high damage resistance threshold facilitate the acquisition of ultrashort pulse with higher frequency [31]. Thus, the research to date has tended to focus on new materials with large nonlinear coefficient, such as graphene (Im*χ*^(3)^ 10^−13^ esu), single-walled carbon nanotubes (Im*χ*^(3)^ 10^−11^ esu), transition metal dichalcogenides (Im*χ*^(3)^ 10^−14^ esu), and topological insulators (Im*χ*^(3)^ 10^−9^ esu) rather than designing new structured devices [32–34]. Of particular concern is that nano materials usually need to be combined with macromolecular organic compound to form a composite film in order to realize the saturable absorption function [29, 35]. Unfortunately, the poor resistance of macromolecular organic compounds to high temperature and high power leads to a limitation of the harmonic order of the fiber laser, which in turn limits the high repetition frequency. Recent work by researchers has established that the harmonic order is also limited by the modulation depth of the SA [36]. Large modulation depth contributes to achieve higher order harmonics. Of course, the harmonic order is not necessarily higher, the larger the modulation depth is. Q-switching can be caused due to too large modulation depth [37]. Looking at the above factors, the key to achieving high repetition frequency pulse output is that the saturable absorption device has high damage resistance threshold, large nonlinearity, and modulation depth.

It has previously been observed that the one-dimensional asymmetric photonic crystal with defect layer (1D-APCDL) has exactly the above characteristics. 1D-APCDL is the new structured optical device designed by inserting a two-dimensional material layer into the original periodically arranged dielectric layer of a one-dimensional photonic crystal. It is usually based on the preparation of the coating process entirely or the use of polymer organic compounds for spin coating [38, 39]. According to the previous introduction, it is clearly impractical to use polymer organic compounds. In addition, it is difficult to prepare high-performance doped two-dimensional materials based on physical vapor deposition process because the films prepared from the targets are mostly polycrystalline and lose good nonlinear absorption properties.

Here we report, a novel film structure of 1D-APCDL which can be used in fiber laser was designed to replace the conventional macromolecular organic compound-nanomaterial film structure. The 1D-APCDL was fabricated by the combination of coating technology and self-assembly technology. Specifically, a bismuth telluride-based topological material with large modulation depth was designed through doping. On this basis, the structure of 1D-APCDL was designed based on finite element simulation (Comsol Multiphysics). As a new type of film structure, it not only improves the damage threshold of the SA, but also improves its nonlinearity significantly. We obtained a stable femtosecond pulse output with a repetition frequency of up to 3.82 GHz (99th order) in the experiment. As far as we know, it is the highest repetition frequency of topological insulator-based saturable absorber in ring fiber laser currently. It is worth mentioning that the 1D-APCDL structure has a wide applicability, the similar effects can also be achieved by other saturable absorption materials.

## 2 Synthesis and characterization of Bi_1.6_Sb_0.4_Te_3_ nanosheets

**Figure 1:**
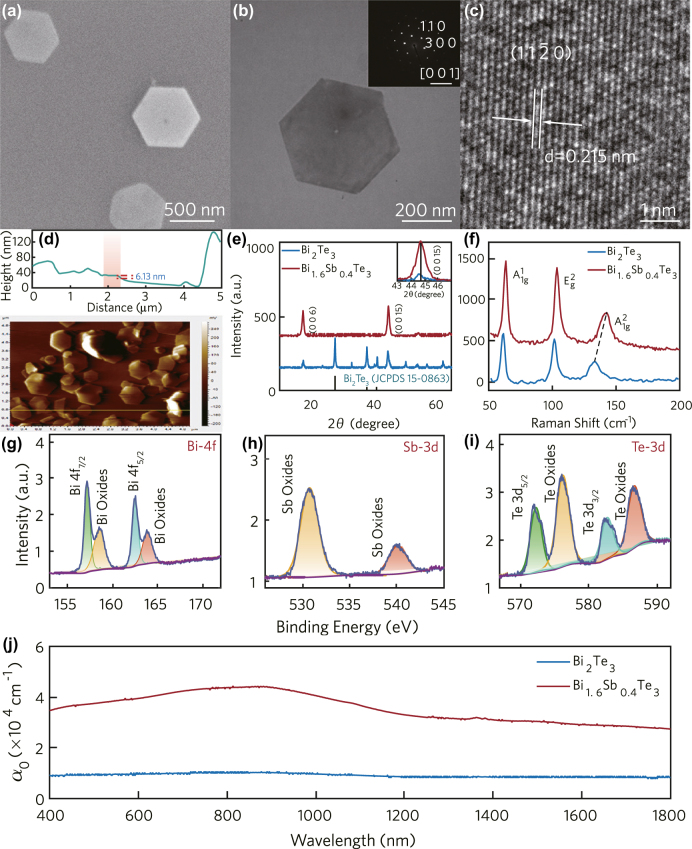
Characterization of Bi_1.6_Sb_0.4_Te_3_. (a) SEM, (b) TEM (the inset is SAED pattern), (c) HRTEM, (d) AFM, (e) XRD patterns, and (f) Raman spectra; XPS spectra corresponding to the (g) Bi 4f, (h) Sb 3d, (i) Te-3d, and (j) absorption spectra.

It has been proved that the modulation depth of topological insulator can be improved effectively by doping in our previous research [40]. Therefore, we prepared Bi_1.6_Sb_0.4_Te_3_ by solvothermal synthesis in the first step. The specific preparation method is consistent with our previous work. As shown in Figure 1(a), the sample has a regular hexagonal shape with a lateral size of about 0.5 µm. The HRTEM (Figure 1(c)) shows that the lattice fringe spacing of sample is 0.215 nm, which conforms to the hexagonal lattice structure (1 1 $\bar{2}$ 0) plane spacing. The result of AFM shows that the lateral size of the sample is about 500 nm, and the longitudinal size is about 6.13 nm. The morphology and size correspond well to the result of SEM. In order to confirm that the sample has good crystallinity, the XRD of Bi_2_Te_3_ and Bi_1.6_Sb_0.4_Te_3_ were analyzed, as shown in Figure 1(e). For non-doped samples, it can be well matched with the rhombohedral Bi_2_Te_3_ standard card, and there is no impurity phase. In addition, the angle of the strongest diffraction peak (0, 0, and 15) of Bi_1.6_Sb_0.4_Te_3_ shifts to a larger angle compared with Bi_2_Te_3_. The crystal structure and molecular vibration can be analyzed effectively by the Raman spectroscopy. As shown in Figure 1(f), the three vibration peaks of Bi_1.6_Sb_0.4_Te_3_ all move to higher frequencies compared with Bi_2_Te_3_. It is attributed to the fact that the electronegativity of Sb atoms is greater than that of Bi atoms. In addition, the result is in consonance with the XRD result: the unit cell size becomes smaller with Sb atom doping. XPS was applied to determine the doping ratio of Bi_1.6_Sb_0.4_Te_3_. As shown in Figure 1(g)–(i), not only the electron peaks of the corresponding elements were observed in the sample, but also the electron peaks after oxidation of the elements were observed, which indicated that the samples were oxidized during the transfer process. The actual proportions of the three elements can be obtained through Gaussian fitting of the narrow scan peaks of the sample, which are 33.9%, 6.6%, and 59.5%, respectively. It is extremely close to the design proportions. The steady-state optical response of the sample is measured by an ultraviolet spectrophotometer, from 400–1800 nm. The sample is dissolved in ethylene glycol evenly. The steady-state optical response of the sample is obtained after removing the absorption curve of the glycol solution, as shown in Figure 1(j). Obviously, the absorption coefficient of the doped sample is much greater than that of Bi_2_Te_3_.

**Figure 2:**
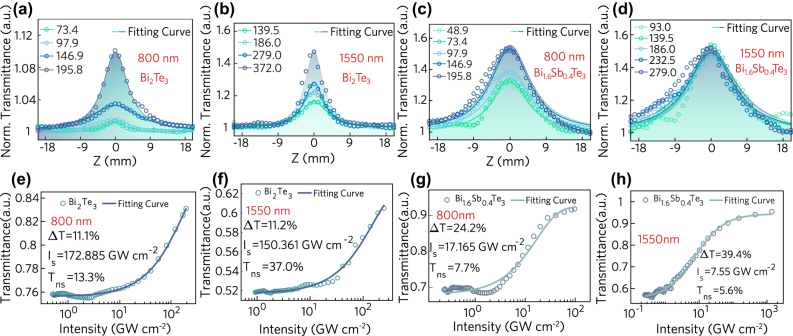
The non-linear results of Bi_2_Te_3_ and Bi_1.6_Sb_0.4_Te_3_. (a)–(b) Z-scan results of Bi_2_Te_3_ at 800 nm and 1550 nm (the unit of numbers in the legend is GW cm^−2^), (c)–(d) Z-scan results of Bi_1.6_Sb_0.4_Te_3_ at 800 nm and 1550 nm, (e)–(f) the modulation depth curve of Bi_2_Te_3_, (g)–(h) the modulation depth curve of Bi_1.6_Sb_0.4_Te_3_.

The broadband nonlinear absorption characteristic of the sample is analyzed by the widely used Z-scan technique. The open-aperture z-scan information of Bi_2_Te_3_ and Bi_1.6_Sb_0.4_Te_3_ at 800 nm and 1550 nm were measured in order to analyze the non-linear absorption performance of the doped sample. As shown in Figure 2(a)–(d), the normalized transmittance is the largest at the focal point of the *z*-axis. It can be attributed to the fact that the intensity of the pump laser increases as the distance from the sample to the focal point decreases, so the sample exhibits saturated absorption effect. In addition, the absorption characteristics of the sample are different under different wavelengths of pump light. The z-scan curve of the sample can be fitted according to the following equation [41]:

(1) $$T\left(z\right)=1-\beta I_{0}L_{\text{eff}}/\left(2^{\frac{3}{2}}\left(1+z^{2}/z_{0}^{2}\right)\right)$$

where *β* is the non-linear absorption coefficient of the sample, *I*_0_ is the peak intensity at *z* = 0, *z*_0_ is the Rayleigh range of the sample at *z* = 0, *L*_eff_ is the effective thickness of the sample, which can be expressed as: $L_{\text{eff}}=\frac{1-{\text{e}}^{-\alpha_{0}L}}{\alpha_{0}}$ , where *α*_0_ is the linear absorption coefficient of the sample, *L* is the actual thickness of the sample. Since the third-order nonlinear coefficient of the sample has an important influence on the saturated absorption, it is necessary to obtain the imaginary part of the third-order nonlinear polarizability, which can be expressed as: $Im\chi^{\left(3\right)}=\frac{2\varepsilon_{0}c^{2}n_{0}^{2}}{3\omega }\beta$ , where *ɛ*_0_ is the vacuum dielectric constant, *c* is the speed of light, and *n*_0_ is the refractive index of the material in isopropanol (*n*_0_ ≈ 1.37) [42]. In order to avoid the difference caused by linear absorption, the figure of merit FOM is used as an important evaluation index, where $\text{FOM}=\left|Im\chi^{\left(3\right)}\right|/\alpha_{0}$ . The open-aperture z-scan fitting curve of sample can be obtained according to Eq. (1), as shown in Figure 2.

The modulation depth curve of the sample can be obtained by extracting the z-scan data, and the curve can be fitted by the following equation [28]:

(2) $$T\left(z\right)=1-\frac{\Delta T}{1+\frac{I}{I_{\text{s}}}}-T_{\text{ns}}$$

where Δ*T*, *I*_s_, and *T*_ns_ are the modulation depth, saturation intensity and non-saturable loss, respectively. The corresponding fitting curve is shown in Figure 2(e)–(h). It is obvious that the saturable absorption characteristics are observed for both materials at 800 nm and 1550 nm, which indicate that the materials prepared have strong nonlinearity in a wide band. It is worth noting that, the modulation depth of the doped sample is significantly higher than that of Bi_2_Te_3_ comparing the modulation depth curves of the two materials at 1550 nm. It can be attributed to the rearrangement of electrons after doping, in which the microscopic mechanism has been explained in our past work [40]. In addition, the calculation results of various optical coefficients of the samples are shown in Table 1. The nonlinear absorption coefficient *β*, the imaginary part of the third-order nonlinear polarizability Im*χ*^(3)^ and FOM of Bi_1.6_Sb_0.4_Te_3_ at 1550 nm are an order of magnitude higher than Bi_2_Te_3_, which indicates that the doped sample is more suitable as a high-performance saturable absorber.

**Table 1:** Nonlinear performance of materials and 1D-APCDL at 800 nm and 1550 nm.

	Sample	*α*_0_(10^4^ cm^−1^)	I_s_(GW cm^−2^)	*β*(10^2^ cm GW^−1^)	Im*χ*^(3)^(10^−11^ esu)	FOM(10^−13^ esu cm)
800 nm	Bi_2_Te_3_	1.02	172.89	−0.076 ± 0.002	5.61 ± 0.17	0.06 ± 0.002
	Bi_1.6_Sb_0.4_Te_3_	4.37	17.17	−12.68 ± 0.0138	939.99 ± 10.21	2.15 ± 0.023
1550 nm	Bi_2_Te_3_	0.84	150.36	−0.36 ± 0.002	51.45 ± 2.16	0.61 ± 0.026
	Bi_1.6_Sb_0.4_Te_3_	2.98	7.55	−6.92 ± 0.063	994.13 ± 9.03	3.34 ± 0.03
	1D-APCDL	4.07	0.885	−39.15 ± 0.48	5622.62 ± 69.23	13.804 ± 0.17

**Figure 3:**
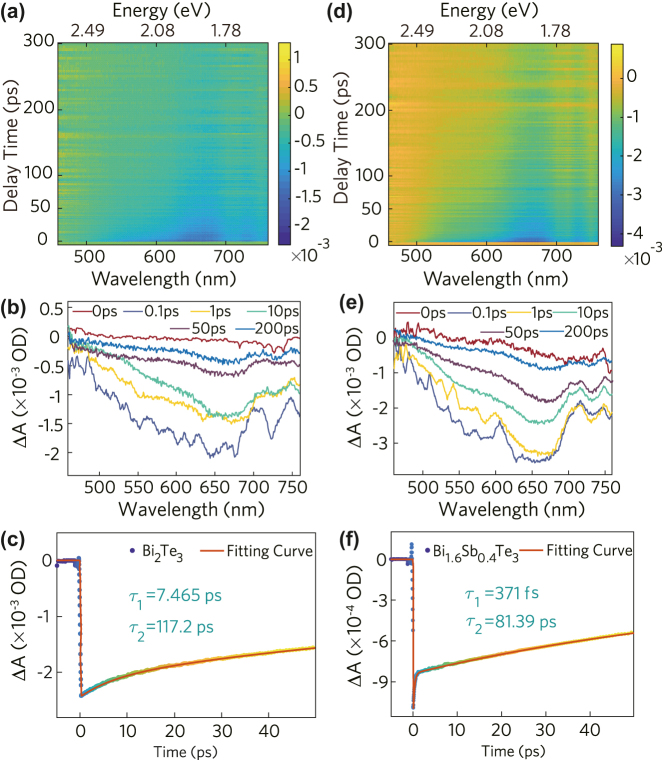
Carrier dynamics analysis of Bi_2_Te_3_ and Bi_1.6_Sb_0.4_Te_3_. (a) 2D mapping of the transient absorption spectrum from 460 to 760 nm, (b) transient absorption spectra of Bi_2_Te_3_, (c) principal dynamic curve of Bi_2_Te_3_, (d) 2D mapping of the transient absorption spectrum from 460 to 760 nm, (e) transient absorption spectra of Bi_1.6_Sb_0.4_Te_3_, (f) principal dynamic curve of Bi_1.6_Sb_0.4_Te_3_.

The relaxation time of carriers is of great significance to the performance of femtosecond lasers. While the sample is exposed to high optical intensity, it may cause ground state bleaching, stimulated emission, and excited state absorption, which constitute the total nonlinear absorption. Therefore, the photo-induced carrier dynamics of Bi_2_Te_3_ and Bi_1.6_Sb_0.4_Te_3_ were further studied by femtosecond resolved transient absorption spectrometer. The photon-induced bleach of the two samples in the probe band is clearly observed at the pump wavelength of 400 nm, as shown in Figure 3. It is worth noting that the ΔA of the two samples is less than 0, which indicates that only the ground state bleaching and stimulated emission of the photons occurred in the sample under the action of the pump light. The carrier dynamics curve at 650 nm can be fitted according to a double exponential function, and the fitting equation is as follows [43]:

(3) $$\Delta A=A_{1}\exp\left(-t/\tau_{1}\right)+A_{2}\exp\left(-t/\tau_{2}\right)$$

where *τ*_1_ and *τ*_2_ are the fast relaxation time and slow relaxation time of samples, respectively. For Bi_1.6_Sb_0.4_Te_3_, it can be analyzed from the carrier dynamics curve that electrons are first excited to the conduction band and leave holes in the valence band. The cooling kinetics of it can be divided into two processes: the electrons first reach thermal equilibrium in 371 fs in the conduction band, and further, the hot electrons cool down to the valence band and recombine with holes in 81.39 ps. The fast and slow relaxation times are 7.465 ps and 117.2 ps for Bi_2_Te_3_, respectively. Apparently, the slow relaxation *τ*_2_ time of the doped sample is significantly lower than that of the undoped sample. It is attributed to the introduction of impurities and defects in the doped sample. A recombination center level in the forbidden band may be formed by these impurities and defects, which can further promote the recombination of non-equilibrium carriers [44]. Therefore, the prepared Bi_1.6_Sb_0.4_Te_3_ can be further used to achieve a higher performance saturable absorption device.

## 3 Design, preparation and characterization of 1D-APCDL

**Figure 4:**
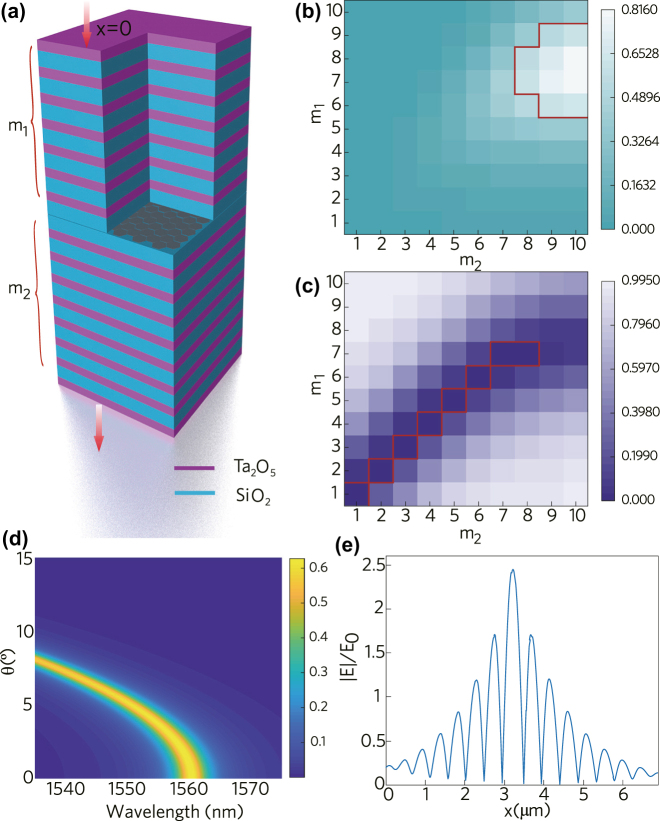
Finite element simulation of 1D-APCDL. (a) 1D-APCDL structure model, (b)–(c) absorptivity and reflectivity distribution map of 1D-APCDL with different periods, (d) absorptance distribution of 1D-APCDL under different wavelengths and incident angles, (e) electric field intensity distribution at different positions.

The 1D-APCDL we designed is composed of SiO_2_, Ta_2_O_5_, and Bi_1.6_Sb_0.4_Te_3_. The structure was shown in Figure 4(a). The refractive index of SiO_2_ and Ta_2_O_5_ are *n*_1_ = 1.444 and *n*_2_ = 2.07, respectively. The center of the transport defect state of the designed asymmetric quarter-wavelength one-dimensional photonic crystal is located at the central wavelength position of the fiber laser, i.e., 1562 nm. For the SiO_2_ on both sides of the defect layer, according to the photonic crystal equation:

(4) $$2n_{1}d_{1}=\lambda /2$$

where *n*_1_ and *d*_1_ are the refractive index and geometric thickness of SiO_2_, respectively. *λ* is the center wavelength. The geometric thickness of SiO_2_ can be calculated to be 270.4 nm according to Eq. (4). Furthermore, according to the general equation of photonic crystals:

(5) $$n_{1}d_{1}+n_{2}d_{2}=\lambda /2$$

where *n*_2_ and *d*_2_ are the refractive index and geometric thickness of Ta_2_O_5_, respectively. The geometric thickness of Ta_2_O_5_ can be calculated to be 188.7 nm according to Eq. (5). For the defect layer, the Bi_1.6_Sb_0.4_Te_3_ nanosheets are single-layer structure and the thickness of it can be seen from Figure 1(d), which is about 6 nm.

Based on the finite element simulation software COMSOL, the optical performance of the photonic crystal is analyzed according to the above data. A 1D-APCDL structure is established in the wave optics module. For the defect layer, the refractive index curve and extinction coefficient curve of Bi_1.6_Sb_0.4_Te_3_ can be obtained based on the VASP simulation results, and the optical properties of the photonic crystal can be obtained accurately based on it. In order to meet the requirements of mode-locked fiber lasers, the designed photonic crystal not only has high absorptivity, but also has low reflectivity. Therefore, the absorptivity and reflectivity of different combinations of layers can be obtained by changing the number of upper layer periods *m*_1_ and the number of lower layer periods *m*_2_ in COMSOL. The absorptivity of the photonic crystal is shown in Figure 4(b). Inside the red line is the combination of high absorptivity. Similarly, inside the red line of Figure 4(c) is the combination of low reflectivity. The specific period number of photonic crystals with high absorptivity and low reflectivity can be obtained by taking the overlap of the two parts. Finally, it is concluded that the performance of the photonic crystal is the best when the upper layer has 7 periods and the lower layer has 8 periods. In addition, the absorption characteristic of the photonic crystal at different center wavelengths of light with different incident angles was analyzed, as shown in Figure 4(d). It is obvious that the absorptivity decays rapidly as the incident angle increases for the photonic crystal with center wavelength of 1562 nm. The absorptivity of the photonic crystal is the largest, when the light incident angle is 0° (i.e. the perpendicular incidence). The electric field intensity at different positions in the perpendicular direction of the photonic crystal is analyzed in order to understand the micro-mechanism of absorption enhancement. The electric field intensity curve at different positions in the photonic crystal is shown in Figure 4(e). Apparently, Bi_1.6_Sb_0.4_Te_3_ obtains several times the electric field enhancement at the position of the photonic crystal defect layer, which is the essential reason for the enhanced absorption of 1D-APCDL.

**Figure 5:**
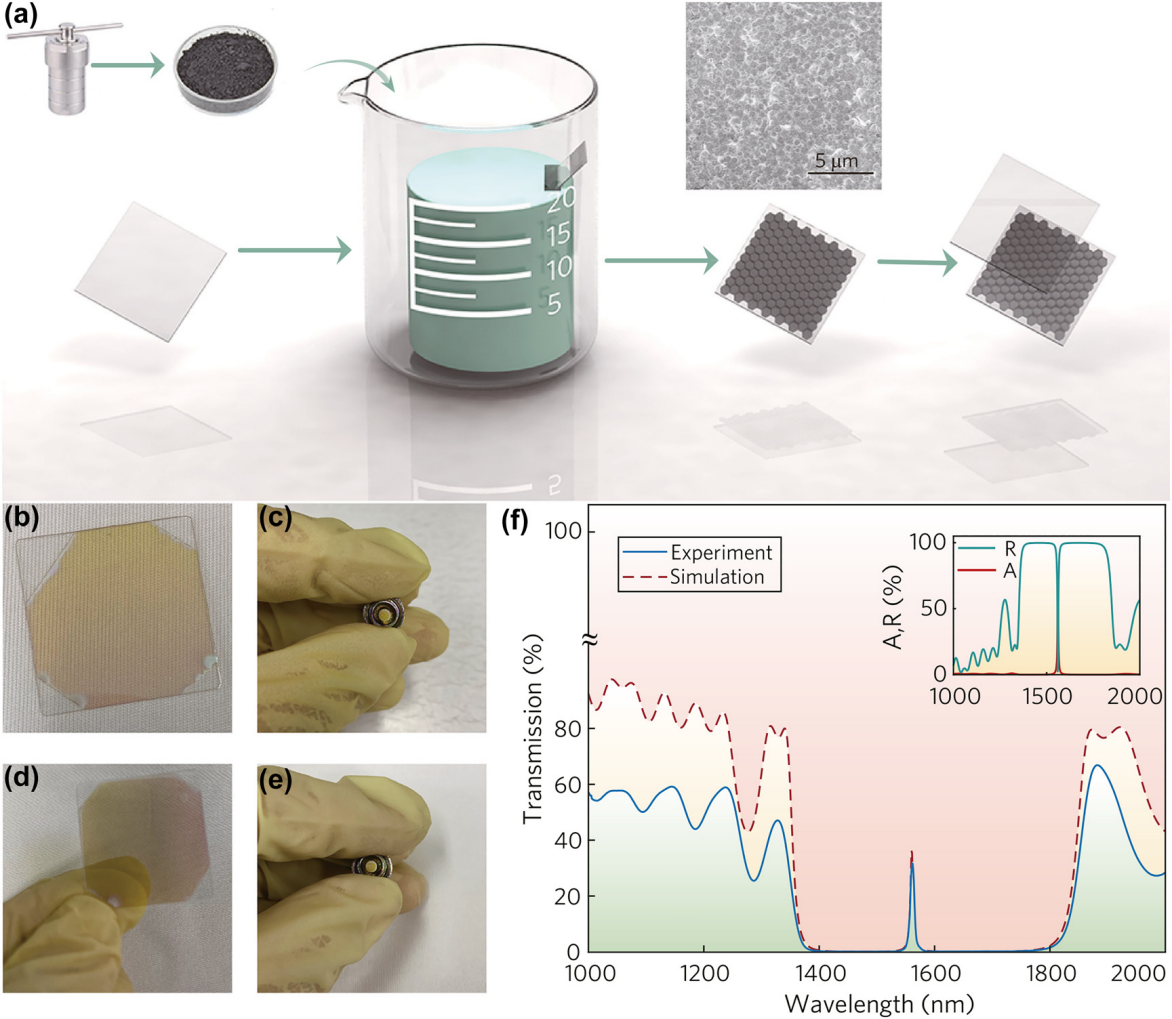
Preparation process of 1D-APCDL. (a) Self-assembly schematic process (the insert shows the self-assembly effect in SEM), (b)–(c) photonic crystal after the first coating, (d)–(e) 1D-APCDL, and (f) simulation and experimental results of transmittance, absorptivity, and reflectivity.

A simple and economical self-assembly method is proposed in order to realize the thin film preparation of single crystal topological insulators, and the corresponding schematic diagram is shown in Figure 5(a). First of all, the silicon glass with thickness of 20 μm was cleaned by acetone, ethanol, and deionized water, respectively. It is worth noting that the power of ultrasonic machine should be lower than 120 W, otherwise the substrate will be easily broken. The cleaned substrate is placed in a fume hood for natural drying and transferred to the Leybold electron beam evaporation machine after the solution on substrate evaporating completely. The Ta_2_O_5_ film and SiO_2_ film are plated cyclically according to the designed parameters. Among them, the Leybold electron beam evaporation machine is first evacuated to a vacuum degree of 3.3 × 10^−5^ Pa. The deposition rate of Ta_2_O_5_ and SiO_2_ are 0.2 nm s^−1^ and 0.6 nm s^−1^, respectively. The half-period taken out photonic crystal was cleaned by acetone, ethanol and deionized water when the coating of the lower part of the film was completed. The prepared Bi_1.6_Sb_0.4_Te_3_ powder was dissolved in deionized water and the concentration was adjusted to 0.13 mmol L^−1^. The solution was placed in an ultrasonic machine and sonicated for 30 min. Furthermore, the self-made Teflon carrier is placed in the beaker, and the coated half-period photonic crystal (the coated surface is facing upwards) is placed in the slot position of the carrier. The beaker was placed in a fume hood for 3 days to complete the self-assembly process. The self-assembled half-period photonic crystal was placed in the Leybold electron beam evaporation machine for secondary coating to complete the preparation of the 1D-APCDL. The coating operation is completed on the cover glass and the end face of the fiber patch cord respectively in order to facilitate testing and application. The photonic crystals before self-assembly are shown in Figure 5(b) and (c), and the prepared photonic crystals are shown in Figure 5(d) and (e). The linear optical absorption performance and the nonlinear absorption optical performance are characterized for the prepared 1D-APCDL respectively. For the linear absorption part, the transmission curve of the photonic crystal is analyzed by the PerkinElmer LAMBDA 1050 spectrophotometer, as shown in Figure 5(f). The blue line is the experimental result and the red line and insert figure are the COMSOL simulation results. Obviously, the photonic crystal has a strong absorption at 1.56 µm. The experimental result is basically consistent with the simulation result.

**Figure 6:**
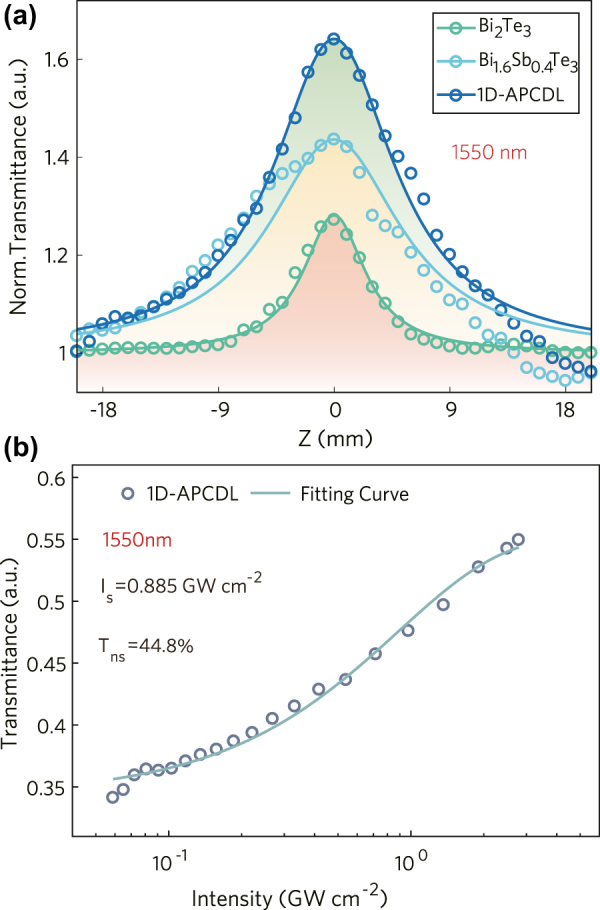
Nonlinear characteristic of 1D-APCDL. (a) Z-scan comparison of three samples, (b) the modulation depth curve of 1D-APCDL.

The non-linear performance of the 1D-APCDL was measured at 1.55 µm by z-scan technique according to the working wavelength of the photonic crystal. The z-scan curves of the three samples are characterized at the same power density in order to compare their performance, as shown in Figure 6(a). The curves show that the nonlinear performance of 1D-APCDL is higher than Bi_2_Te_3_ and Bi_1.6_Sb_0.4_Te_3_. The modulation depth curve of 1D-APCDL is fitted with the z-scan curve. It is obvious that the modulation depth of 1D-APCDL is much higher than that of Bi_2_Te_3_. The results of nonlinear absorption coefficient *β*, third-order nonlinear polarizability Im*χ*^(3)^ and FOM of 1D-APCDL are shown in Table 1. The nonlinear absorption coefficient and third-order nonlinear polarization rate of the prepared photonic crystal are increased by 2 orders of magnitude compared with Bi_2_Te_3_, and the figure of merit is increased by 20 times, which indicates that the 1D-APCDL has excellent nonlinear optical performance.

## 4 High-order harmonic fiber laser with 1D-APCDL

The high-energy pulse will split into multiple pulses in fiber laser if there is an excess of soliton energy, and the repetition frequency will be increased. According to the soliton area theorem, the energy of the pulse can be expressed as Eq. (6) [45]:

(6) $$E_{\text{p}}=\frac{1.1346|\beta_{2,\text{avg}}|}{\Delta \tau \cdot \gamma_{\text{avg}}}$$

where *β*_2,avg_ and *γ*_avg_ are the average group velocity dispersion and average nonlinearity of cavity, respectively. Δ*τ* is the full width at half maximum of the pulse. It is concluded that a ring cavity with low dispersion or high nonlinearity contributed to reducing the average energy of the soliton, and which in turn promotes pulse splitting faster. The 1D-APCDL we designed exactly satisfies the high nonlinearity.

**Figure 7:**
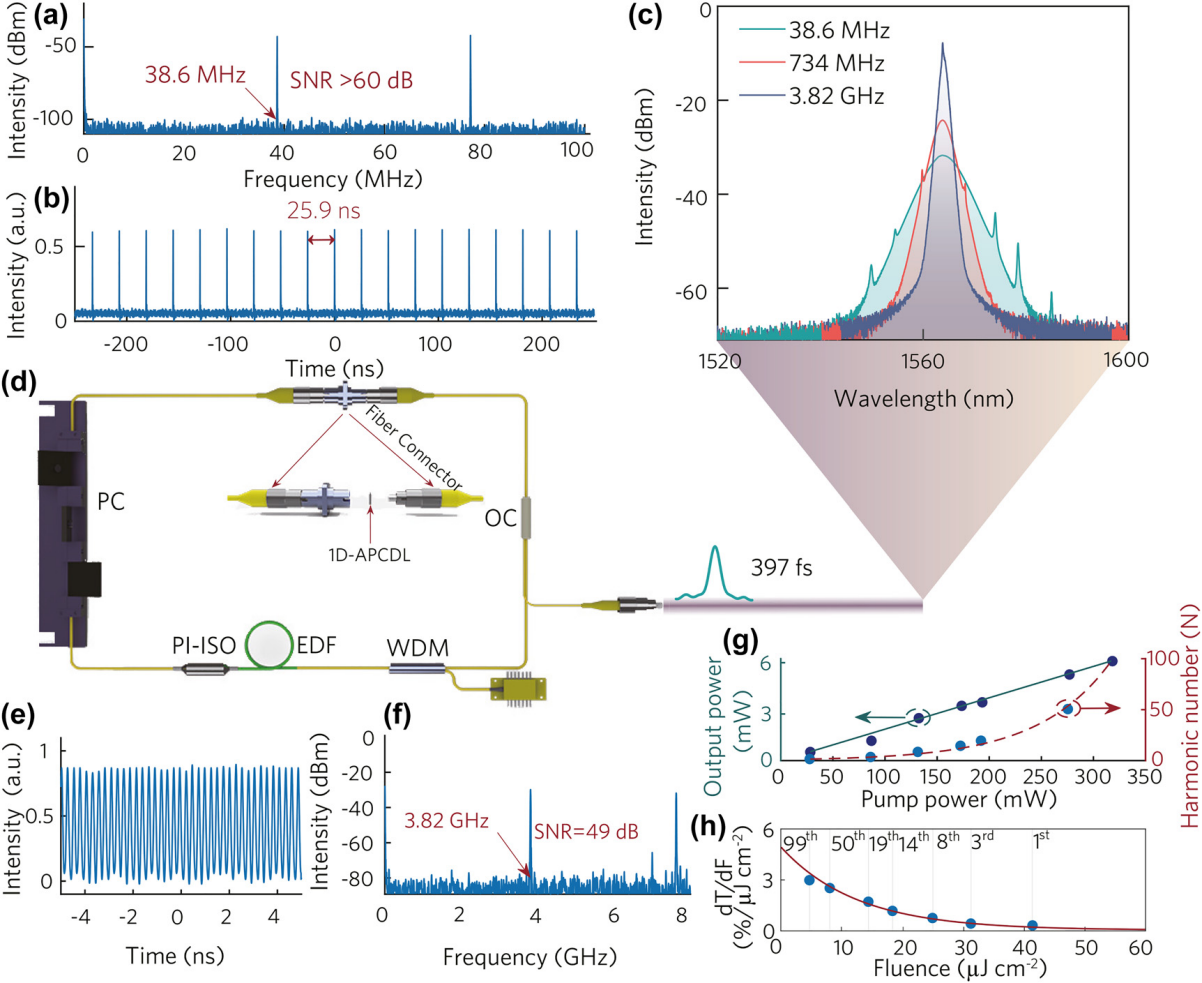
Application of 1D-APCDL in ultrafast fiber laser. (a) RF spectrum (fundamental frequency), (b) pulse train, (c) mode-locked spectra of different orders, (d) cavity schematic, (e) pulse train (99th), (f) RF spectrum, (g) the curve of output power and harmonic order varying with pump power, and (h) first derivative of nonlinear transmittance curve with respect to fluence.

Erbium-doped ring cavity fiber laser is applied in the experiment, the corresponding device is shown in Figure 7(d). The ring cavity used in the experiment includes 500 mW pump source, wavelength division multiplexer (WDM), polarization independent isolator (PI-ISO), polarization controller (PC), 10% output coupler, 30 cm long erbium-doped fiber with a group velocity dispersion parameter (GVD) of 20 ps^2^ km^−1^, and single mode fiber with a GVD of −23 ps^2^ km^−1^. The total length of the ring cavity fiber laser is 5.3 m.

The mode-locked pulse of the fundamental repetition frequency with output power of 0.509 mW is realized in the fiber laser at the pump power of 28.28 mW. The oscilloscope shows that the fundamental repetition frequency is 38.6 MHz, and the interval between adjacent pulses is 25.9 ns, which corresponds well to the cavity length, as shown in Figure 7(a) and (b). The output power at this point is too low to accurately measure the pulse duration by the autocorrelator. The minimum pulse duration at the fundamental frequency was 397 fs in our past experiment [40]. The 3 dB bandwidth of the spectrum is 6.6 nm according to the result recorded by the spectrometer. The RF spectrum is widely used to determine the stability of pulses, and the result shows that the signal-to-noise ratio (SNR) of the fundamental frequency pulse is greater than 60 dB, which indicates that the pulse is robust. In addition, Kelly sidebands are clearly observed on the spectrometer, which is caused by the periodic disturbance of soliton pulses in the laser resonator. There is weak dispersion in this soliton mode-locked fiber laser due to the absence of a dispersion compensating fiber in the ring cavity. The pulse will be subjected to disturbance during the propagation process due to the discrete nature of the dispersion, which causes the soliton and the dispersive wave to propagate together. The quasi-phase matching occurs in the resonant cavity synchronously. The phase difference between the dispersive wave and the soliton is integer times of 2*π* at the specific frequencies. Kelly sidebands will be formed on the spectrum correspondingly [46].

The order of the harmonic is mainly determined by the power of the pump for the harmonic mode locking process. The harmonics of different orders during the pump power increase were observed and recorded. The harmonic pulse output with a repetition frequency of 116 MHz (corresponding to the 3rd order) is observed on the oscilloscope when the pump power reaches 86.7 mW. With further increase in pumping power, the repetition frequencies of the output harmonic pulses are observed to be 309, 541, 734, 1935, and 3820 MHz when the power is 131.99, 172.98, 192.65, 275.99, and 317 mW, respectively. The corresponding high-harmonic mode-locked soliton trains were shown in Figure S1. It should be pointed out that the angle of the polarization controller needs to be adjusted slightly during the process. It can be explained that the strong birefringence effect will occur when light is transmitted in the fiber, and the relative phase shift of the two polarization modes will be induced during the propagation process. The disturbance along the fiber will destroy the matching between the wavenumber and the propagation constants of the two polarization modes because of the vibration of the optical platform which is almost inevitable. Further, the disturbance along the fiber will change slowly, and the light cannot be effectively coupled, which in turn leads the polarization state changes greatly during the propagation process, and destroys the original mode locking conditions ultimately [47]. The pulse spectrums with distinctive features are recorded by the spectrometer, as shown in Figure 7(c). The pulse widths at different repetition rates were 413 fs, 793 fs, and 2.2 ps, which were recorded by the autocorrelator. The corresponding autocorrelation traces and fitting curves were shown in Figure S2. The spectral width gradually decreases as the repetition frequency increases, and the Kelly sideband gradually disappears. The specific reason will be explained later. The RF spectrum shows that the signal-to-noise ratio (SNR) is 49 dB for the 3820 MHz harmonic pulse, which indicates that the pulse is still very robust, as shown in Figure 7(f). The distribution of the pulse in the time-domain is shown in Figure 7(e). To test the long-term stability of fiber laser, the output spectrum was recorded every 3 h under pump power of 317 mW, as shown in Figure S3. The central wavelength and shape of the spectrum maintain good stability during the recording time, even if the pump power has exceeded 300 mW. It is due to the protection of Bi_1.6_Sb_0.4_Te_3_ by Ta_2_O_5_ and SiO_2_ in 1D-APCDL structure. The relationship between the average output power of the pulse and the pump power is shown in Figure 7(g). The harmonic order and output power are increasing as the pump power increases. The harmonic output power is 5.738 mW when the harmonic order reaches 99. In addition, the relationship between pump power and harmonic order is also recorded in Figure 7(g). It is worth noting that the relationship between them is not linear, but in the form of an exponential function. The nonlinearity can be explained by the following: the working point on the saturable absorption curve moves to the direction of low fluence as the order of harmonics increases according to the report by Boguslawski et al. [36]. Therefore, the higher the order of the harmonics, the lower the remaining effective modulation depth of the pulse, which in turn causes the spectral width to gradually decrease, it also be confirmed in Figure 7(c). The pump power range for pulse stable operation gradually becomes smaller as the effective modulation depth decreases, that is, the pulse will split rapidly for high-order pulses when the pump power is slightly increased. It must be pointed out that the splitting will not continue indefinitely because of the limited modulation depth of the saturable absorber. The pulse will not continue to split when the modulation depth is “fully utilized” and meanwhile the fluence of the pulse has also reached the lowest value. The rate of modulation depth change can be described by the first derivative of the saturable absorption curve with respect to the pulse fluence (d*T*/d*F*), the corresponding curve is shown in Figure 7(h). It is obvious that the curve decreases exponentially, which indicates that low fluence has a greater influence than high fluence on the change of modulation depth. It also explains why the input power is exponentially related to the pulse repetition frequency in Figure 7(g) reasonably. Furthermore, it can be concluded that the modulation depth of the saturable absorber should be appropriately increased while reducing the saturation fluence in order to obtain a higher repetition frequency. The 1D-APCDL structure we designed has these characteristics exactly. Therefore, the structure can be further extended to other materials and wavelength, and used in ultrafast harmonic mode-locked fiber lasers.

## 5 Conclusions

In summary, 1D-APCDL is beneficial to obtain higher order harmonic mode-locked pulse. 1D-APCDL with high absorptivity and low reflectivity was designed through COMSOL supported by systematic experiments. It can enhance the absorption effect of the defect layer effectively, improve the nonlinearity of the sample, and reduce the saturated fluence. It is applied to ultrafast fiber lasers to achieve high-order, high-repetition frequency femtosecond pulse output. This work strongly proves that 1D-APCDL can be effectively applied to high repetition frequency pulsed lasers of various wavelengths. It also provides new ideas for the application of material-based nonlinear optical chip in the field of laser processing.

## Supplementary Material

Supplementary Material Details

## References

[1] Pang M., Jiang X., He W. (2015). Stable subpicosecond soliton fiber laser passively mode-locked by gigahertz acoustic resonance in photonic crystal fiber core. *Optica*.

[2] Terra O. (2019). Absolute frequency measurement of the hyperfine structure of the 5s1/2 – 5d3/2 two-photon transition in rubidium using femtosecond frequency comb. *Measurement*.

[3] Fang D., Zazzi A., Mueller J. (2022). Optical arbitrary waveform measurement using silicon photonic slicing filters. *J. Lightwave Technol.*.

[4] Novo C. C., Choudhury D., Siwicki B., Thomson R. R., Shephard J. D. (2020). Femtosecond laser machining of hollow-core negative curvature fibres. *Opt Express*.

[5] Li Q., Wang Q., Li L. (2020). Femtosecond laser-etched MXene microsupercapacitors with double-side configuration via arbitrary on- and through-substrate connections. *Adv. Energy Mater.*.

[6] Ceccarelli F., Atzeni S., Pentangelo C., Pellegatta F., Crespi A., Osellame R. (2020). Low power reconfigurability and reduced crosstalk in integrated photonic circuits fabricated by femtosecond laser micromachining. *Laser Photon. Rev.*.

[7] Jakob S., Pfeifenberger M. J., Hohenwarter A., Pippan R. (2017). Femtosecond laser machining for characterization of local mechanical properties of biomaterials: a case study on wood. *Sci. Technol. Adv. Mater.*.

[8] You K., Yan G., Luo X., Gilchrist M. D., Fang F. (2020). Advances in laser assisted machining of hard and brittle materials. *J. Manuf. Process.*.

[9] Zheng Q., Fan Z., Jiang G. (2019). Mechanism and morphology control of underwater femtosecond laser microgrooving of silicon carbide ceramics. *Opt Express*.

[10] Rebro P. A., Shin Y. C., Incropera F. P. (2002). Laser-Assisted machining of reaction sintered mullite ceramics. *J. Manuf. Sci. Eng.*.

[11] Kerse C., Kalaycıoğlu H., Elahi P. (2016). Ablation-cooled material removal with ultrafast bursts of pulses. *Nature*.

[12] Mishchik K., Bonamis G., Qiao J. (2019). High-efficiency femtosecond ablation of silicon with GHz repetition rate laser source. *Opt. Lett.*.

[13] Gao W., Liu G., Zhang Z. (2018). 44.6fs pulses from a 257MHz Er:fiber laser mode-locked by biased NALM. *Chin. Opt Lett.*.

[14] McFerran J. J., Nenadović L., Swann W. C., Schlager J. B., Newbury N. R. (2007). A passively mode-locked fiber laser at 1.54 μm with a fundamental repetition frequency reaching 2GHz. *Opt Express*.

[15] Nicholson J. W., DiGiovanni D. J. (2008). High-repetition-frequency low-noise fiber ring lasers mode-locked with carbon nanotubes. *IEEE Photon. Technol. Lett.*.

[16] Martinez A., Yamashita S. (2011). Multi-gigahertz repetition rate passively modelocked fiber lasers using carbon nanotubes. *Opt Express*.

[17] Liu X., Pang M. (2019). Revealing the buildup dynamics of harmonic mode-locking states in ultrafast lasers. *Laser Photon. Rev.*.

[18] Nelson L. E., Jones D. J., Tamura K., Haus H. A., Ippen E. P. (1997). Ultrashort-pulse fiber ring lasers. *Appl. Phys. B Laser Opt.*.

[19] Wang Z., Zhan L., Majeed A., Zou Z. (2015). Harmonic mode locking of bound solitons. *Opt. Lett.*.

[20] Liu J.-S., Li X.-H., Guo Y.-X. (2019). SnSe2 nanosheets for subpicosecond harmonic mode-locked pulse generation. *Small*.

[21] Feng J., Li X., Shi Z. (2020). 2D ductile transition metal chalcogenides (TMCs): novel high-performance Ag2S nanosheets for ultrafast photonics. *Adv. Opt. Mater.*.

[22] Tao S., Xu L., Chen G., Gu C., Song H. (2016). Ultra-high repetition rate harmonic mode-locking generated in a dispersion and nonlinearity managed fiber laser. *J. Lightwave Technol.*.

[23] Tamura K., Haus H. A., Ippen E. P. (1992). Self-starting additive pulse mode-locked erbium fibre ring laser. *Electron. Lett.*.

[24] Gumenyuk R. V., Korobko D. A., Zolotovskii I. O. (2020). Stabilization of passive harmonic mode locking in a fiber ring laser. *Opt. Lett.*.

[25] Wang F., Rozhin A. G., Scardaci V. (2008). Wideband-tuneable, nanotube mode-locked, fibre laser. *Nat. Nanotechnol.*.

[26] Grudinin A. B., Gray S. (1997). Passive harmonic mode locking in soliton fiber lasers. *J. Opt. Soc. Am. B*.

[27] Chen H., Gao L., Qin Z. (2020). Recent advances of low-dimensional materials in Mid- and Far-infrared photonics. *Appl. Mater. Today*.

[28] Xu N., Li H., Gan Y. (2020). Zero-dimensional MXene-based optical devices for ultrafast and ultranarrow photonics applications. *Adv. Sci.*.

[29] Guo J., Shi R., Wang R. (2020). Graphdiyne-polymer nanocomposite as a broadband and robust saturable absorber for ultrafast photonics. *Laser Photon. Rev.*.

[30] Ma C., Wang C., Gao B., Adams J., Wu G., Zhang H. (2019). Recent progress in ultrafast lasers based on 2D materials as a saturable absorber. *Appl. Phys. Rev.*.

[31] Jun C. S., Choi S. Y., Rotermund F., Kim B. Y., Yeom D.-I. (2012). Toward higher-order passive harmonic mode-locking of a soliton fiber laser. *Opt. Lett.*.

[32] Guo B., Xiao Q.-l., Wang S.-h., Zhang H. (2019). 2D layered materials: synthesis, nonlinear optical properties, and device applications. *Laser Photon. Rev.*.

[33] Horiuchi N. (2015). Nonlinear opportunities. *Nat. Photonics*.

[34] Botti S., Ciardi R., De Dominicis L., Asilyan L. S., Fantoni R., Marolo T. (2003). DFWM measurements of third-order susceptibility of single-wall carbon nanotubes grown without catalyst. *Chem. Phys. Lett.*.

[35] Chen T., Cheng C., Lin Y. (2019). Optimizing the self-amplitude modulation of different 2-D saturable absorbers for ultrafast mode-locked fiber lasers. *IEEE J. Sel. Top. Quant. Electron.*.

[36] Bogusławski J., Soboń G., Zybała R., Sotor J. (2019). Towards an optimum saturable absorber for the multi-gigahertz harmonic mode locking of fiber lasers. *Photon. Res.*.

[37] Keller U. (1998). Chapter 4 semiconductor nonlinearities for solid-state laser modelocking and Q-switching. *Semiconduct. Semimet.*.

[38] Lova P., Manfredi G., Comoretto D. (2018). Advances in functional solution processed planar 1D photonic crystals. *Adv. Opt. Mater.*.

[39] Zhang S., Shen C., Kislyakov I. M. (2019). Photonic-crystal-based broadband graphene saturable absorber. *Opt. Lett.*.

[40] Song C., Zhang H., Jin L. (2020). Study on the energy band regulation of bi_2−*x*_sb_
*x*
_te_3_ and its application as mode locking material in low gain ultrafast fiber laser. *Adv. Opt. Mater.*.

[41] Guo J., Huang D., Zhang Y. (2019). 2D GeP as a novel broadband nonlinear optical material for ultrafast photonics. *Laser Photon. Rev.*.

[42] Sani E., Dell’Oro A. (2016). Spectral optical constants of ethanol and isopropanol from ultraviolet to far infrared. *Opt. Mater.*.

[43] Xie Z., Zhang F., Liang Z. (2019). Revealing of the ultrafast third-order nonlinear optical response and enabled photonic application in two-dimensional tin sulfide. *Photon. Res.*.

[44] Dumke W. P. (1957). Spontaneous radiative recombination in semiconductors. *Phys. Rev.*.

[45] Yan P., Lin R., Ruan S., Liu A., Chen H. (2015). A 2.95 GHz, femtosecond passive harmonic mode-locked fiber laser based on evanescent field interaction with topological insulator film. *Opt Express*.

[46] Gordon J. P. (1992). Dispersive perturbations of solitons of the nonlinear Schrödinger equation. *J. Opt. Soc. Am. B*.

[47] Schreiber T., Röser F., Schmidt O. (2005). Stress-induced single-polarization single-transverse mode photonic crystal fiber with low nonlinearity. *Opt Express*.

