# Supplementary material for: Gigahertz femtosecond laser-by a novel asymmetric one-dimensional photonic crystal saturable absorber device with defect layer
Source: Nanophotonics. 2022 May 10;11(12):2939–51. doi: 10.1515/nanoph-2022-0145 (PMC11501611; doi:10.1515/nanoph-2022-0145)
Supplement: Supplementary file 1 — Supplementary Material Details [file j_nanoph-2022-0145_suppl.docx]

**Supplementary Information for**

**Gigahertz Femtosecond Laser-By a Novel Asymmetric One-Dimensional Photonic Crystal Saturable Absorber Device with Defect Layer**

Chun-Yu Song†, Hua-Long Chen†, Yong-Jie Wang, Liang Jin, Ying-Tian Xu, Lin-Lin Shi, Yong-Gang Zou, Xiao-Hui Ma, Yu-Feng Song, Cong Wang, Ya-Ting Zhang, Ja-Hon Lin, He Zhang*, Han Zhang*, and Jian-Quan Yao*

†These authors contributed equally to this work.

**Chun-Yu Song**†**, Yong-Jie Wang, Liang Jin, Ying-Tian Xu, Lin-Lin Shi, Yong-Gang Zou, Xiao-Hui Ma, He Zhang,** State Key Laboratory of High Power Semiconductor Lasers, Changchun University of Science and Technology, Changchun

130022, P. R. China, e-mail: zhanghe@cust.edu.cn

**Hua-Long Chen**†**, Yu-Feng Song, Cong Wang, Han Zhang,** International Collaborative Laboratory of 2D Materials for Optoelectronics Science and Technology of Ministry of Education, Institute of Microscale Optoelectronics, Shenzhen University, Shenzhen 518060, P. R. China, e-mail: hzhang@szu.edu.cn

**Chun-Yu Song**†**, Ya-Ting Zhang, Jian-Quan Yao,** School of Precision Instruments and Opto-Electronics Engineering, Tianjin University, Tianjin 300072, P. R. China, e-mail: jqyao@tju.edu.cn

**Ja-Hon Lin,** Institute of Electro-Optical Engineering, National Taipei University of Technology, Taipei 10608, Taiwan

*The preparation Process of 1D-APCDL*: First of all, the silicon glass with thickness of 20μm was cleaned by acetone, ethanol and deionized water, respectively. It is worth noting that the power of ultrasonic machine should be lower than 120 W, otherwise the substrate will be broken easily. The cleaned substrate is placed in a fume hood to dry naturally and transferred to the Leybold electron beam evaporation machine after the solution on substrate evaporating completely. The Ta_2_O_5_ and SiO_2_ film are plated cyclically according to the designed parameters. Among them, the Leybold electron beam evaporation machine is first evacuated to a vacuum degree of 3.3 × 10^-5^ Pa. The deposition rate of Ta_2_O_5_ and SiO_2_ are 0.2 nm s^-1^ and 0.6 nm s^-1^, respectively. The half-period taken out photonic crystal was cleaned by acetone, ethanol and deionized water when the coating of the lower part of the film was completed. The prepared Bi_1.6_Sb_0.4_Te_3_ powder was dissolved in deionized water and the concentration was adjusted to 0.13 mmol L^-1^. The solution was placed in an ultrasonic machine and sonicated for 30 minutes. Furthermore, the self-made Teflon carrier is placed in the beaker, and the coated half-period photonic crystal (the coated surface is facing upwards) is placed in the slot position of the carrier. The beaker was placed in a fume hood for 3 days to complete the self-assembly process. The self-assembled half-period photonic crystal was placed in the Leybold electron beam evaporation machine for secondary coating to complete the preparation of the 1D-APCDL.

*Transient Absorption Experiment*: The broadband non-degenerate TA spectrometer is applied to investigate the carrier dynamic processes of Bi_2_Te_3_ and Bi_1.6_Sb_0.4_Te_3_. In the setup, the pump laser (400 nm) is generated by nonlinear frequency conversion, and repeated frequency of pump laser is 500 Hz. The nonlinear frequency conversion processes include the sum frequency, frequency doubling, beat frequency, etc. The broadband probe super-continuum laser (460-760 nm) is generated by irradiating the 800 nm laser on various crystals and repeated frequency of probe laser is 1000 Hz. The probe laser irradiates the detector, which generates differential absorbance signal. The total probe window is 8 ns.

*Z-Scan Experiment*: The ultrafast pulses with different wavelengths were divided to two components by a 50:50 beam splitter. One was set as the reference light and collected by a photodetector (PD1). The other one was injected into the sample and the transmitted light was shaped by a convex lens and collected by the other detector (PD2). When moving forward the computer-controlled stage where the sample was fixed on from -Z to Z, the absorption variation versus the position can be monitored basing on the power variation at PD2. The transmittance of sample is calculated with values of the two photodetectors. Before measurement, a 0.5 mm ZnSe thin crystal was used to align the optical path.


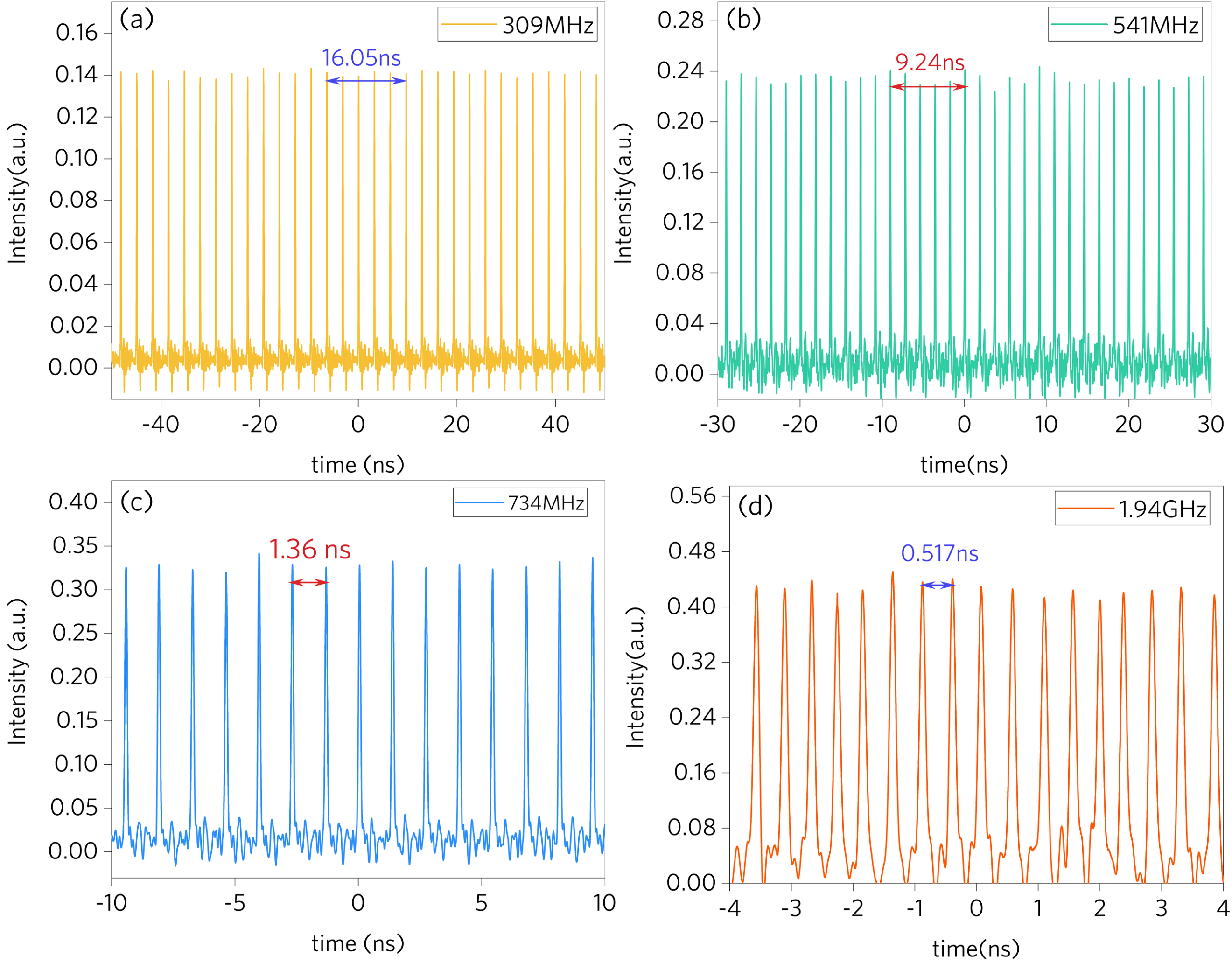


**Figure S1.** High-harmonic mode-locked soliton trains. (a) 8th at 309 MHz, (b) 14th at 541 MHz, (c) 19th at 734 MHz, (d) 50th at 1.94GHz.


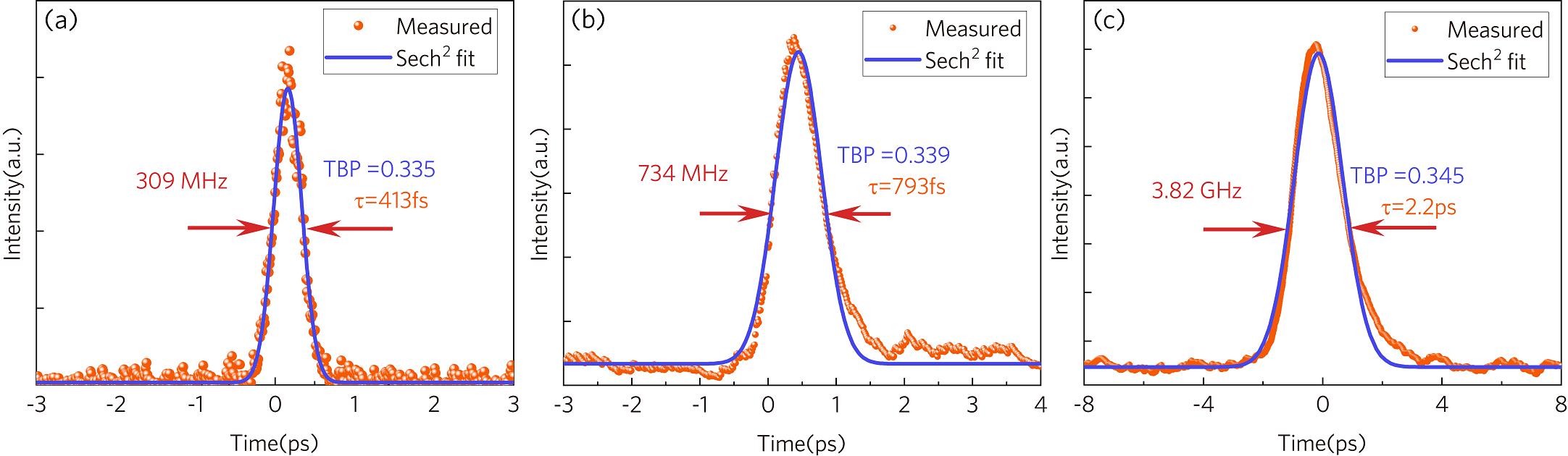


**Figure S2.** The autocorrelation trace of harmonic mode-locked pulses of different orders. (a) 8th at 38.6 MHz, (b) 19th at 734 MHz, (c) 99th at 3.82 GHz.


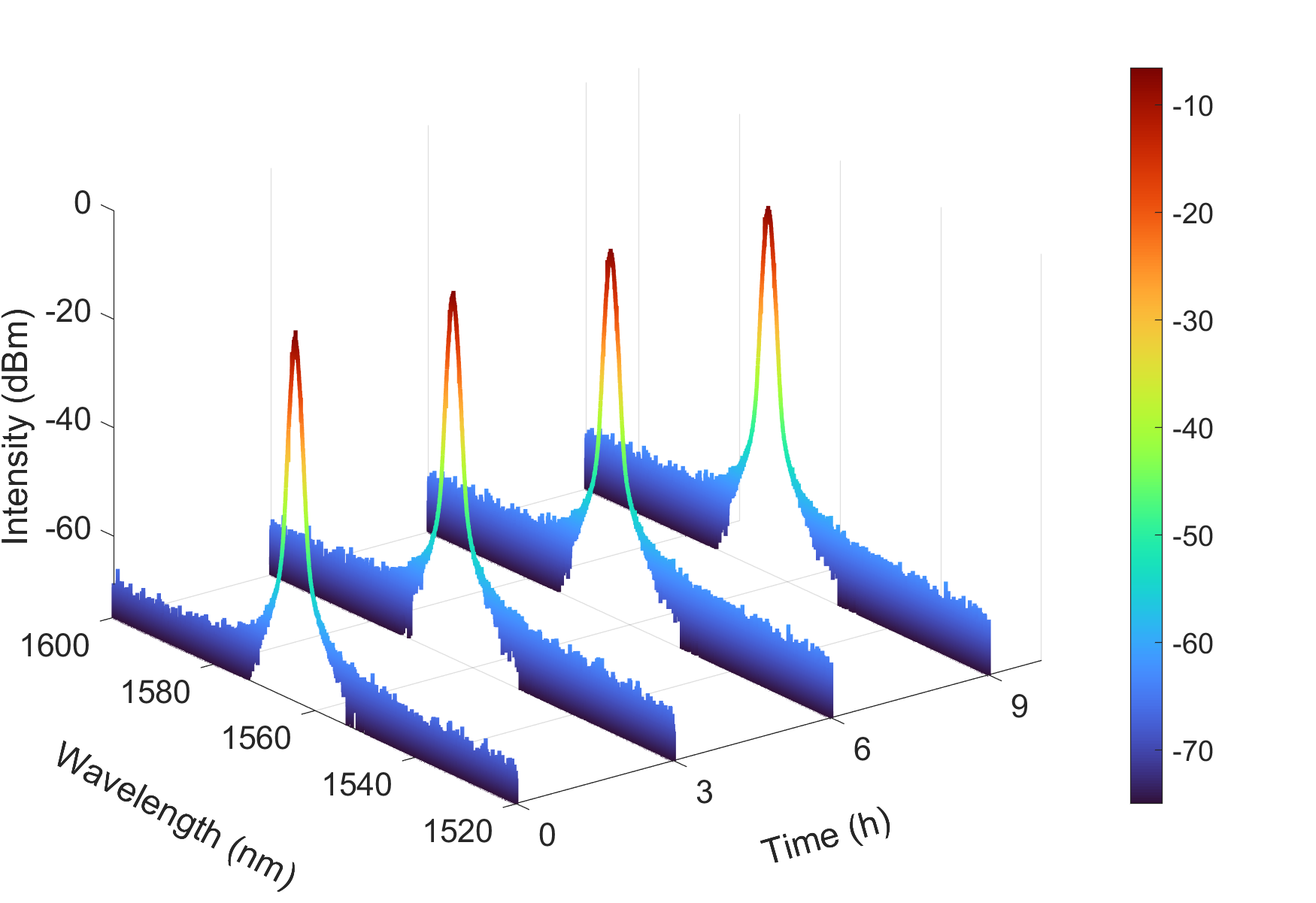


**Figure S3.** Long-term stability of the high-harmonic output spectrum measured under different time.
